# Fucoidan from *Fucus vesiculosus* Protects against Alcohol-Induced Liver Damage by Modulating Inflammatory Mediators in Mice and HepG2 Cells

**DOI:** 10.3390/md13021051

**Published:** 2015-02-16

**Authors:** Jung Dae Lim, Sung Ryul Lee, Taeseong Kim, Seon-A Jang, Se Chan Kang, Hyun Jung Koo, Eunsoo Sohn, Jong Phil Bak, Seung Namkoong, Hyoung Kyu Kim, In Sung Song, Nari Kim, Eun-Hwa Sohn, Jin Han

**Affiliations:** 1 Department of Herbal Medicine Resource, Kangwon National University, Gangwon-do 245-905, Korea; E-Mails: ijdae@kangwon.ac.kr (J.D.L.); noddle1@hanmail.net (T.K.); 2 College of Medicine, Cardiovascular and Metabolic Disease Center and Department of Health Sciences and Technology, Graduate School of Inje University, Inje University, Busan 614-735, Korea; E-Mails: lsr1113@inje.ac.kr (S.R.L.); estrus74@gmail.com (H.K.K.); microvirus@hanmail.net (I.S.S.); phykimnr@inje.ac.kr (N.K.); 3 Department of Life Science, Gachon University, Seongnam 461-701, Korea; E-Mails: white7068@daum.net (S.A.J.); sckang73@gachon.ac.kr (S.C.K.); 4 Department of Medicinal and Industrial Crops, Korea National College of Agriculture and Fisheries, Hwasung 445-760, Korea; E-Mail: dewykoo@gmail.com; 5 Division of Information Analysis Research, Korea Institute of Science and Technology Information, KISTI, Seoul 130-741, Korea; E-Mail: essohn@kisti.re.kr; 6 The Clinical Center for Bio-industry, Semyung University, Jecheon, 390-711, Korea; E-Mail: jpbak77@gmail.com; 7 Department of Physical Therapy, Kangwon National University, Gangwon-do 245-711, Korea; E-Mail: seungnk@kangwon.ac.kr

**Keywords:** fucoidan, transforming growth factor beta 1, cyclooxygenase-2, alcohol, liver

## Abstract

Fucoidan is an l-fucose-enriched sulfated polysaccharide isolated from brown algae and marine invertebrates. In this study, we investigated the protective effect of fucoidan from *Fucus vesiculosus* on alcohol-induced murine liver damage. Liver injury was induced by oral administration of 25% alcohol with or without fucoidan (30 mg/kg or 60 mg/kg) for seven days. Alcohol administration increased serum aspartate aminotransferase and alanine aminotransferase levels, but these increases were suppressed by the treatment of fucoidan. Transforming growth factor beta 1 (TGF-β1), a liver fibrosis-inducing factor, was highly expressed in the alcohol-fed group and human hepatoma HepG2 cell; however, the increase in TGF-β1 expression was reduced following fucoidan administration. Treatment with fucoidan was also found to significantly reduce the production of inflammation-promoting cyclooygenase-2 and nitric oxide, while markedly increasing the expression of the hepatoprotective enzyme, hemeoxygenase-1, on murine liver and HepG2 cells. Taken together, the antifibrotic and anti-inflammatory effects of fucoidan on alcohol-induced liver damage may provide valuable insights into developing new therapeutics or interventions.

## 1. Introduction

Alcohol consumption is the world’s third largest risk factor for disease and disability, since alcohol consumption can serve as a causal factor in 60 types of diseases [1]. Alcoholic liver disease (ALD) has been becoming an important public health issue widely around the world, including Korea, due to the increasing consumption of alcoholic beverages [2]. Excessive and consistent alcohol exposure leads to hepatic steatosis, hepatitis, cirrhosis and progressive fibrosis. Chronic liver diseases, such as hepatic fibrosis and cirrhosis, are considered as major health problems worldwide [3,4].

Alcohol is mainly metabolized in the liver, and toxic metabolites, such as acetaldehyde, excessive homocysteine and toxic lipid species, are formed during alcohol metabolism [3,5]. The liver is highly susceptible to fibrotic remodeling from ethanol detoxification [4]. Therefore, chronic and excessive ethanol consumption leads to fatty liver in more than 90% of chronic alcohol abusers [5]. In some populations, more severe forms of alcoholic liver diseases, including alcoholic fibrosis and cirrhosis, are observed [5,6].

It has been suggested that inflammation-mediated fibrosis is largely attributable to fibrogenic cytokines released by infiltrating immune cells, as well as paracrine and autocrine stimulation of hepatic cells [3,4]. Among various inflammatory mediators, transforming growth factor-β1 (TGF-β1) is one of the most powerful profibrotic cytokines [7,8], and thus, blocking TGF-β1 activity by natural inhibitors represents a valid and logical strategy to combat hepatic fibrosis [8,9]. Cyclooxygenase-2 (COX-2), which catalyzes prostaglandin production from arachidonic acid, is induced by lipopolysaccharides (LPS), peptidoglycans and alcohol [10]. The upregulation of inducible COX-2 expression is an important aspect of inflammatory responses and participates in the augmentation of allyl alcohol-induced liver injury by LPS [11] and the progression of hepatic fibrosis [12]. Augmented production of nitric oxide (NO) is also attributed to the development of hepatic injury [6,13]. Alcohol consumption induces a significant increase in hepatic NO generation, which can be achieved by nitric oxide synthase (NOS), especially through inducible NOS (iNOS). Increases in NO generation may therefore be an early indicator of ethanol-induced liver damage [6]. Hemeoxygenase-1 (HO-1) is an inducible form of the rate-limiting enzyme involved in heme catabolism. In preclinical models of tissue injury, HO-1 has been shown to confer cellular protection through inhibition of apoptosis, inflammation and cell proliferation [14,15]. To combat alcohol-induced liver damage, strategies to strengthen the protective effects of molecules, such as HO-1, and the development of new ways to reduce liver-damaging signals may be favorable treatment options.

Fucoidan, which can be found in brown seaweed extracts (e.g., *Ascophyllum nodosum* and *Fucus vesiculosus*), is a polyanionic macromolecule composed predominantly of sulfated fucose moieties and possesses highly branched polysaccharides [16,17]. Fucoidans derived from almost all species appear to lack toxicity *in vitro* and *in vivo* [18]. However, the means of fucoidan uptake, tissue distribution and final metabolic fate are not well understood. A pivotal study has suggested that fucoidan could be distributed in the rat liver following its oral uptake [19]. Recently, numerous biological activities of fucoidan, including anticoagulant, antithrombotic, antitumor, antiviral, anticomplement and anti-inflammatory effects, have been extensively studied [17,18,20,21]. However, the biological effects of fucoidans can have high variation due to the source of purification, molecular weight, sulfation of fucoidan [16] and delivery route [18]. These known biological activities of fucoidan have been investigated for therapeutic uses against injury, infection, chronic inflammation, fibrosis and neuronal damage [18].

Alcoholic liver disease in humans is difficult to treat, since relatively little is known about the molecular mechanisms involved in its development. In addition, the habit of alcohol consumption is an addictive and self-inflicting condition [5]. Abstinence from alcohol is the first line treatment for alcohol liver disease, but in many cases, it becomes difficult for individuals to remain abstinent [5]. In severe conditions, corticosteroids, pentoxifylline or anti-tumor necrosis factor (TNF) agents are the choice of treatment, but sometimes, these therapeutic agents may cause severe side effects. Thus, there is an urgent need to develop novel therapeutics or preventative interventions against alcoholic liver damage.

Several recent publications suggest that orally-delivered fucoidan has a protective potential against nonalcoholic liver damage by attenuating fibrosis [18,22,23,24], but the mechanisms of antifibrotic action of fucoidan from *Fucus vesiculosus* on alcohol-induced liver damage are not well studied. In line with this concept, the protective role of fucoidan against alcohol-related liver damage was investigated through measurement of the protein expression of TGF-β1, COX-2, HO-1 and iNOS in the livers of alcohol-fed mice and *in vitro* in HepG2 cells.

## 2. Results and Discussion

### 2.1. Changes in Organ Weight in the Presence or Absence of Fucoidan for Alcohol-Fed Mice

Chronic ethanol feeding studies in rodents using either *ad libitum* feeding or intragastric infusion models have significantly enhanced our understanding of the pathogenesis of alcoholic liver disease [2]. Here, we used the intragastric feeding model to induce liver damage. Alcoholic liver disease results from the dose- and time-dependent consumption of alcohol, and thus, the amount of ingested alcohol and its intensity could be carefully controlled through an intragastric feeding model. In addition, the major route of ethanol delivery in humans is clearly oral, and thus, this model is more reliable for understanding alcohol-induced liver pathophysiology.

**Figure 1:**
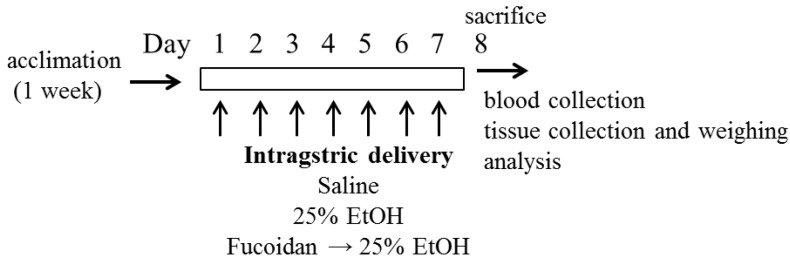
Experimental design.

In this study, alcohol was used as an insult for induced hepatic dysfunction [6]. Mice intragastrically received 25% alcohol (w/v) daily (5 g/kg body weight) with fucoidan (0~60 mg/kg) for one week (Figure 1). At the end of the experiment, the liver, spleen and thymus weights were taken and summarized as relative weight in Table 1. All animals had survived. In the alcohol-treated group, the liver was more enlarged with a slightly brighter color than that of the control. Alcohol-fed mice showed increased liver weight, but thymus weight was significantly decreased (vehicle 0.24% ± 0.01% *vs.* alcohol group 0.21% ± 0.01%, *p* < 0.05). It has been suggested that alcohol causes thymus atrophy, possibly through enhancing thymocyte death [25,26]. As shown in Table 1, the alcohol-mediated decrease in thymus weight was inhibited by fucoidan administration. Administration of fucoidan alone augmented the organ weights of liver, spleen and thymus (Table 1). It is unclear why fucoidan administration alone led to increases in the organ weights; however, this effect may be related to the nutritional support or cell protective effects observed in mice.

**Table 1:** Effect of fucoidan on tissue weights in alcohol-fed mice. Mice were administered alcohol with or without fucoidan daily for 1 week. At the end of the experiment, isolated liver, spleen and thymus were weighed and expressed as a percentage of animal weight (BW; body weight). Each group contains 6 mice. ^#^ *p* < 0.05 *vs.* vehicle group; * *p* < 0.05 *vs.* alcohol-fed group.

	Vehicle	25% (w/v) Alcohol			Fucoidan 30 mg/kg	Fucoidan 60 mg/kg
		-	Fucoidan 30 mg/kg	Fucoidan 60 mg/kg		
	(*n* = 6)	(*n* = 6)	(*n =* 6)	(*n = 6*)	(*n* = 6)	(*n* = 6)
BW (g)	32.7 5 ± 0.92	34.15 ± 0.73	33.60 ± 0.70	33.85 ± 1.20	34.01 ± 1.00	34.45 ± 0.67
Liver (%)	5.13 ± 0.16	5.24 ± 0.11 ^#^	5.65 ± 0.23 *	5.52 ± 0.12 *	5.59 ± 0.05 ^#^	5.31 ± 0.03 ^#^
Spleen (%)	0.27 ± 0.02	0.27 ± 0.09	0.27 ± 0.01	0.32 ± 0.02 *	0.35 ± 0.06 ^#^	0.32 ± 0.01 ^#^
Thymus (%)	0.24 ± 0.01	0.21 ± 0.01 ^#^	0.23 ± 0.09 *	0.23 ± 0.03 *	0.24 ± 0.08 ^#^	0.25 ± 0.13 ^#^

### 2.2. Protective Effects of Fucoidan on Serum Aspartate Aminotransferase and Alanine Aminotransferase in Alcohol-Induced Murine Liver Damage

**Figure 2:**
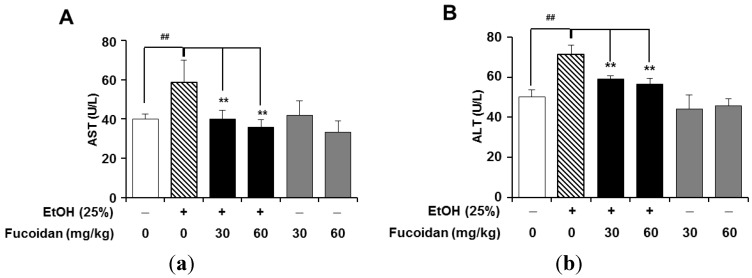
Effect of fucoidan on alcohol-induced liver damage. (**A**) Serum aspartate aminotransferase (AST); (**B**) serum alanine aminotransferase (ALT). **^##^** *p* < 0.01 *vs.* vehicle (*n* = 6); ** *p* < 0.01 *vs.* alcohol-fed group (*n* = 6).

To determine the alcohol-induced liver damage, serum aspartate aminotransferase (AST/GOT) and alanine aminotransferase (ALT/GPT) were determined by the colorimetric method. ALT/GPT is almost exclusively found in the liver. When the liver tissue is diseased or damaged, additional AST and ALT are released into the bloodstream, which increases their activities. Thus, measuring serum levels of AST or ALT is a valuable tool in the diagnosis of liver damage [27]. As shown in Figure 2, the group treated with fucoidan alone did not show any differences in serum levels of AST (vehicle 40.0 ± 2.56 U/L *vs.* alcohol group 58.68 ± 11.12 U/L, *p* < 0.05) and ALT (vehicle 50.0 ± 3.56 U/L *vs.* alcohol group 71.16 ± 4.71 U/L, *p* < 0.05). Alcohol-fed mice showed marked increases in both AST and ALT, which reflect liver damage, but these increases were highly suppressed in the presence of fucoidan. Based on these results, the increased liver weight in the group treated with fucoidan alone may not be derived from liver damage (Table 1), but related to a beneficial effect of fucoidan on liver.

### 2.3. Fucoidan Suppresses the Alcohol-Induced Expression of TGF-β1 in the Murine Liver and HepG2 Cells

**Figure 3:**
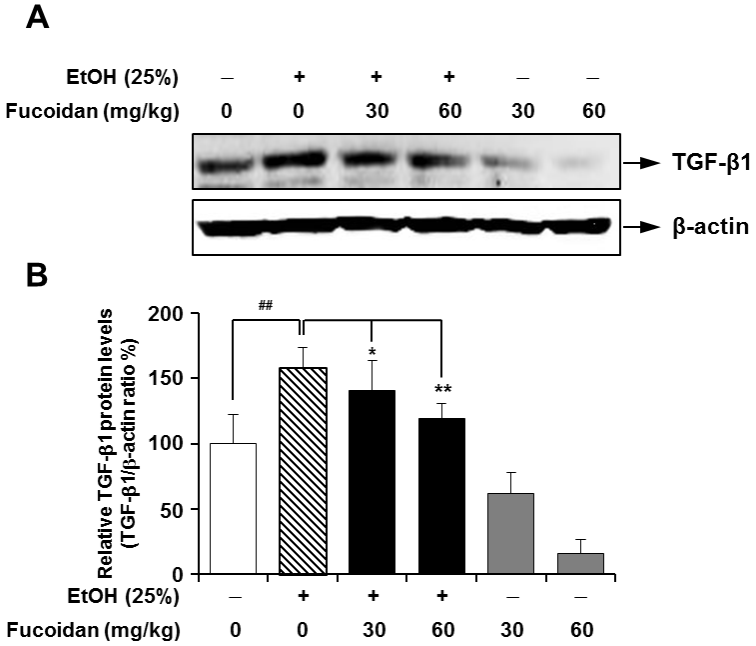
Effect of fucoidan on alcohol-induced protein levels of transforming growth factor (TGF)-β1 in mice livers. (**A**) Representative immunoblot images of TGF-β1 and the loading control β-actin; (**B**) densitometry quantification of TGF-β1 expression normalized to β-actin. ^##^ *p* < 0.05 *vs.* vehicle (*n* = 6); ** *p* < 0.5 *vs.* alcohol-fed group (*n* = 6).

**Figure 4:**
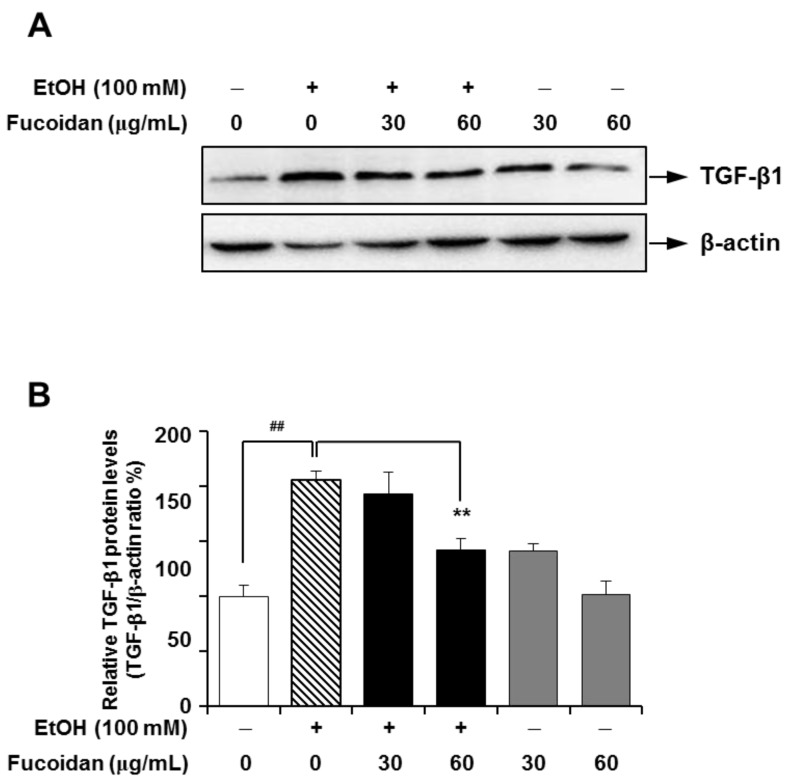
Effect of fucoidan on alcohol-induced protein levels of TGF-β1 in HepG2 cells. (**A**) Representative immunoblot images of TGF-β1 and the loading control β-actin; (**B**) densitometry quantification of TGF-β1 expression normalized to β-actin. **^##^** *p* < 0.05 *vs.* vehicle (*n* = 6); ** *p* < 0.5 *vs.* alcohol-treated group (*n* = 6).

TGF-β1 plays a pivotal role in the induction and maintenance of matrix overproduction, which can be characterized in fibrogenesis [7,8,28], and blocking TGF-β1 activity has proven effective against the fibrotic response to injury in various organs [29]. In line with a previous report [30], alcohol caused a marked increase in the expression of TGF-β1, but fucoidan treatment on alcohol-fed-mice attenuated the expression of TGF-β1 (Figure 3). This inhibitory effect of fucoidan on alcohol-induced TGF-β1 upregulation was further tested in HepG2 cells, which is a suitable cell line for the study of polarized human hepatocytes [30]. As shown in Figure 4, the expression level of TGF-β1 in HepG2 cells was markedly increased following exposure to alcohol, and this increase was also inhibited in the presence of fucoidan*.* Based on Figure 3 and Figure 4, fucoidan may have a general inhibiting activity on TGF-β1 expression, since fucoidan alone also reduced TGF-β1 expression in murine liver and HepG2 cells.

### 2.4. Fucoidan Suppresses NO Production Following Exposure to Alcohol

**Figure 5:**
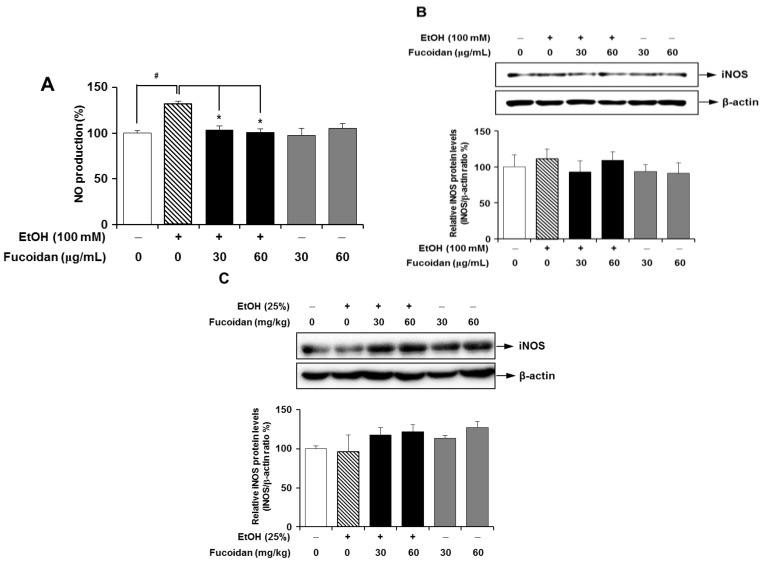
Effect of fucoidan on nitric oxide production and protein expression levels of inducible nitric oxide synthase (iNOS) following alcohol exposure. (**A**) Nitric oxide (NO) production in HepG2 cells. Representative immunoblots and densitometry quantification of iNOS expression in HepG2 (**B**) and murine liver (**C**), both normalized to β-actin expression. # *p* < 0.05 *vs.* vehicle (*n* = 6); * *p* < 0.05 *vs.* alcohol-treated group (*n* = 6).

NO can act like a double-edged sword. It can either mediate beneficial responses or act deleteriously as an inflammatory mediator [31]. Previously, it had been reported that alcohol consumption increases NO production in the rat liver [13]. Thus, the modulatory effect of fucoidan on the ethanol-mediated production of NO was evaluated in HepG2 cells. As shown in Figure 5A, NO was significantly increased in HepG2 cells following exposure to alcohol, and this increase in NO production was strongly attenuated in the presence of fucoidan. NO production following exposure to alcohol can be augmented by increasing enzyme activity or protein expression levels of iNOS [13]. When protein expression levels of iNOS were measured in mouse liver and HepG2 cells, there were no significant changes in iNOS expression (Figure 5B,C). It is unclear why the expression level of iNOS was unchanged, even though NO production was increased after exposure to alcohol. It can therefore be speculated that the production of NO was not mediated by increased iNOS expression. However, augmentation of iNOS activity without changes in protein level is possible [13]. In the present study, fucoidan suppressed the alcohol-induced NO production in hepatocytes, indicating that fucoidan may provide another beneficial activity against alcohol-induced liver inflammatory responses.

### 2.5. Fucoidan Suppresses the Expression Level of COX-2

**Figure 6:**
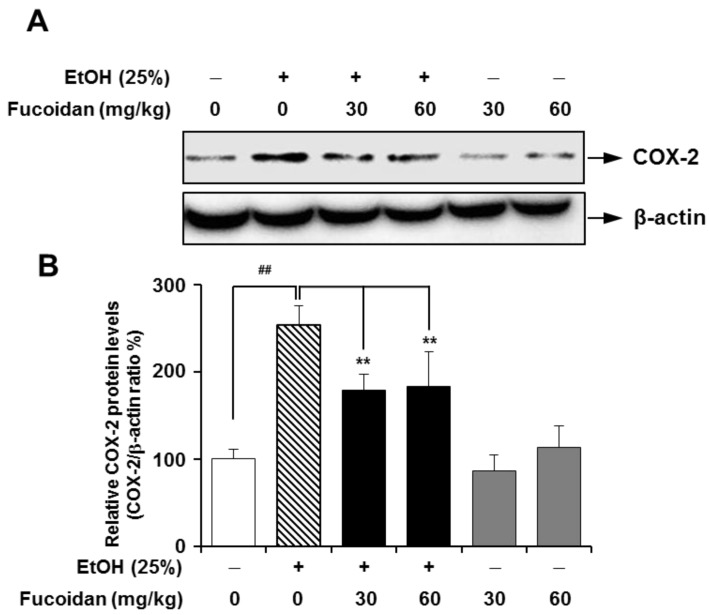
Effect of fucoidan on alcohol-induced expression levels of cyclooxygenase (COX)-2 in murine liver. (**A**) Representative immunoblot images of COX-2 and β-actin; (**B**) quantification of COX-2 expression normalized to β-actin. ^##^ *p* < 0.01 *vs.* vehicle (*n* = 6); ** *p* < 0.01 *vs.* alcohol-fed group (*n* = 6).

**Figure 7:**
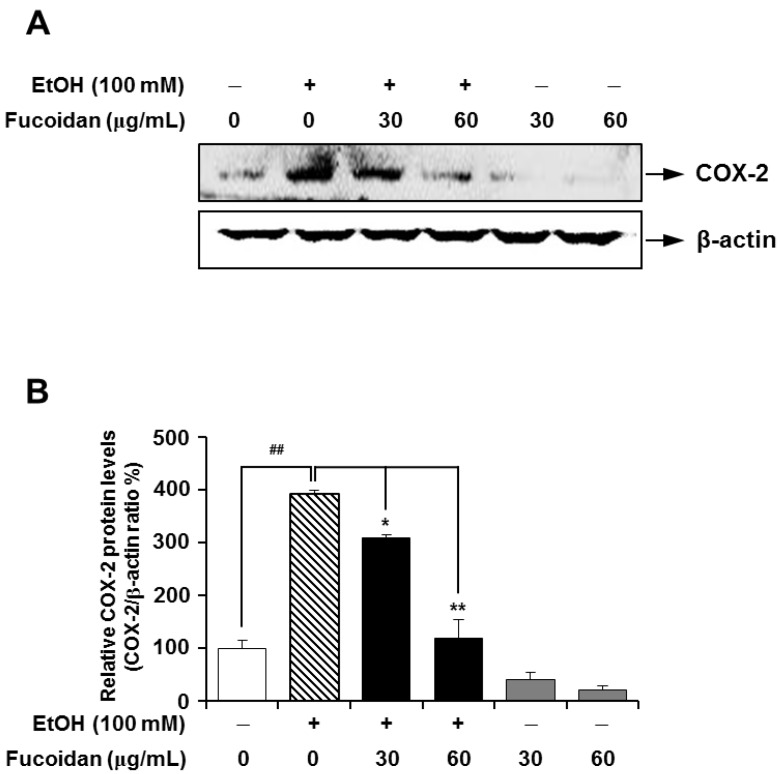
Effect of fucoidan on alcohol-induced expression levels of COX-2 in HepG2 cells. (**A**) Representative immunoblot images of COX-2 and the loading control β-actin; (**B**) quantification of COX-2 expression normalized to β-actin. ^##^ *p* < 0.01 *vs.* vehicle (*n* = 6); * *p* < 0.05 and ** *p* < 0.01 *vs.* alcohol-treated group (*n* = 6).

COX is a key enzyme in the biosynthetic pathway for prostaglandin. Unlike constitutively-expressed COX-1, COX-2 can be upregulated in liver inflammation, and the subsequent production of eicosanoids is an important contributor to liver injury [32]. Additionally, COX-2 may play a role in the progression of hepatic fibrosis, and higher levels of COX-2 expression were observed in a more advanced stage of fibrosis [12]. The inflammatory response is triggered not only by ethanol itself, but also by Gram-negative bacteria through increased intestinal permeability caused by alcohol in the gut [33]. In lipopolysaccharide (LPS)-challenged rats, selective inhibition of COX-2 protected against LPS-induced enhancement of allyl alcohol hepatotoxicity [11]. In our experimental setting, expression levels of COX-2 were markedly higher in the alcohol-treated group (Figure 6). Alcohol-induced upregulation of COX-2 was strongly attenuated in the presence of fucoidan. The inhibitory effect of fucoidan on increased COX-2 was more apparent in alcohol-treated HepG2 cells (Figure 7). The decreased COX-2 expression effects of fucoidan against alcohol insult may also be favorable towards treating alcohol-induced inflammatory liver damage.

### 2.6. Fucoidan Increases the Expression Levels of HO-1

Some investigators have provided the anti-oxidative properties of fucoidan. Kang *et al.* (2008) reported that fucoidan exerts anti-oxidative effects by increasing anti-oxidative enzymes, such as superoxide dismutase (SOD) and glutathione peroxidase (GPx), against CCl_4_-induced liver injury [34]. Hong *et al.* (2011) also reported that fucoidan increased SOD and GPx in the dimethylnitrosamine-induced liver fibrosis model [35]. Moreover, they suggested that the anti-oxidative effect of fucoidan might be mediated by an increase of Nrf2 and its subsequent pathway mediators.

**Figure 8:**
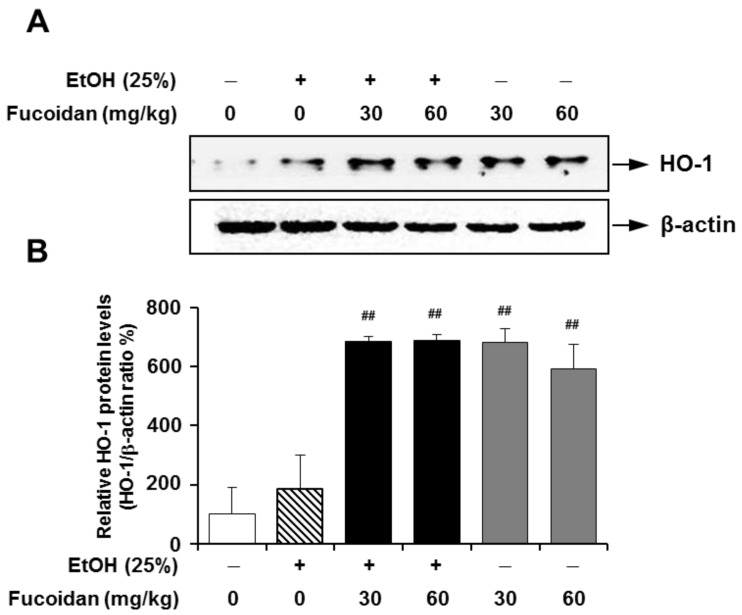
Effect of fucoidan on alcohol-induced expression levels of heme oxygenase (HO)-1 in murine liver. (**A**) Representative immunoblot images of HO-1 and the loading control β-actin; (**B**) quantification of HO-1 expression normalized to β-actin. **^##^** *p* < 0.05 *vs.* vehicle (*n* = 6).

**Figure 9:**
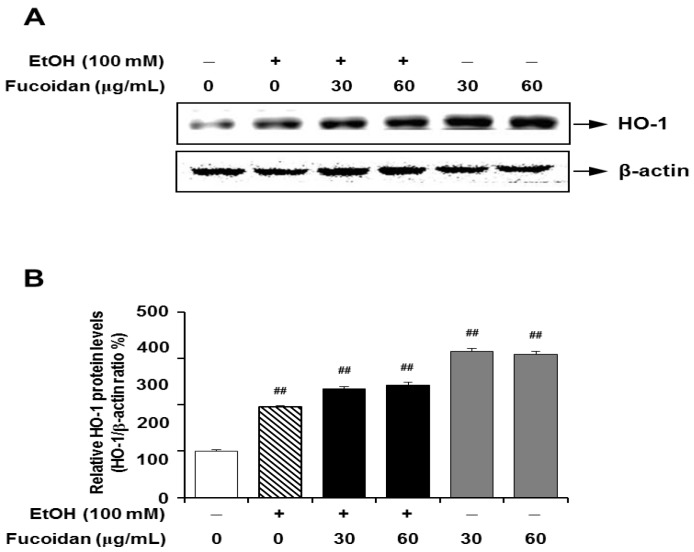
Effect of fucoidan on alcohol-induced expression levels of HO-1 in HepG2 cells. (**A**) Representative immunoblot images of HO-1 and β-actin; (**B**) quantification of HO-1 expression normalized to β-actin. **^##^** *p* < 0.05 *vs.* vehicle (*n* = 6).

Recently, several studies have suggested that the HO-1/nuclear respiratory factor-2 (Nrf-2) pathway confers protective effects against ethanol-, carbon tetrachloride (CCl_4_)- and/or diallyl disulfide-induced oxidative stresses [36,37,38]. HO-1 participates in the rate-limiting step in the heme degradation pathway and maintenance of iron homeostasis. HO-1 acts as a low-molecular-weight stress protein that confers cytoprotection against cell death in various models of lung and vascular injury by inhibiting apoptosis, inflammation and cell proliferation [14]. Induction of HO-1 contributes to inhibiting liver inflammatory mediators, including iNOS and COX-2 through the regulation of JAK-2/STAT-1 signals [39]. Recent studies suggested that the induction of HO-1 prevents ethanol-induced inflammation in the liver via increasing its downstream mediator, carbon monoxide [40]. Carbon monoxide is one of the important regulators of signal pathways that regulate hepatic inflammatory responses, such as MAPKs and Egr-1 [41]. As shown in Figure 8, the expression level of HO-1 was not changed by alcohol exposure, but fucoidan supplementation increased the expression levels of HO-1 in the presence or absence of alcohol in mice liver. The effect of fucoidan on increased expression of HO-1 was also shown in HepG2 cells (Figure 9). However, unlike the alcohol-exposed murine liver, HepG2 cells treated with alcohol alone showed increased HO-1 expression when compared to the control. This discrepancy may be related to different physiological conditions between the *in vivo* and *in vitro* model systems. Although the present study did not directly address the mechanisms of HO-1 induction by fucoidan, these molecular mechanisms might be involved in the process.

## 3. Experimental Section

### 3.1. Animals

Male, 5-week-old BALB/c mice were purchased from Korea Laboratory Animal Co. (Daejeon, Korea) and were allowed to acclimatize for 7 days prior to experiments. The animals were maintained under standard laboratory conditions: temperature of 21 ± 2 °C, relative humidity of 50% ± 5% and a normal photoperiod (12 h dark, 12 h light). The procedures for the experiments and animal care protocol were approved by the Animal Care and Use Committee of Kangwon National University and conformed to the Guide for the Care and Use of Laboratory Animals by the National Institutes of Health (NIH Publication No. 85–23).

### 3.2. Reagents

Extra-pure ethanol (EtOH) was purchased from Sigma (St. Louis, MO, USA). Unless indicated otherwise, chemicals were purchased from Sigma. Antibodies were purchased from Cell Signaling Technology (Beverly, MA, USA).

### 3.3. Fucoidan Preparation

Fucoidan (purity; >95%) extracted from *Fucus vesiculosus* [42] was purchased from Sigma-Aldrich (St. Louis, MO, USA) and dissolved in distilled water. This commercial fucoidan was comprised of a wide spectrum of fucans, ranging from typical fucoidans (major components) containing mainly fucose, sulfate and no uronic acid, to low sulfate-containing heteropolysaccharide-like fucans (minor components). When the QCL-1000 Chromogenic Limulus Amebocyte Lysate End-Point Assay (Lonza, Walkersville, MD, USA) was conducted as described in a previous report [43], the endotoxin level of 10 mg/mL fucoidan preparation was less than 0.1 EU/mL.

### 3.4. Treatment with Alcohol and Fucoidan on Mice and Measurement of Serum ALT, AST and Organ Weight

Alcohol-mediated liver damage was performed based on a previous report [44]. After acclimation, mice were randomly divided into 6 groups with 6 mice in each group, as follows: (1) mice that received an equal volume of saline orally through gavage; (2) mice treated with 5 g/kg body weight of 25% EtOH w/v; (3) mice treated with EtOH plus 30 mg/kg body weight of fucoidan; (4) mice treated with EtOH plus 60 mg/kg body weight of fucoidan; (5) mice treated with 30 mg/kg body weight of fucoidan; and (6) mice treated with 60 mg/kg body weight of fucoidan via oral gavage for 7 days (Figure 1). An appropriate dosing volume 0.5 mL of EtOH and fucoidan was determined after weighing the animal daily. Gavage was performed with 18–20 gauge feeding tubes about 1.5 inches in length with a rounded tip. Intragastric delivery of EtOH or fucoidan was carefully performed by well-trained researcher to minimize animal stress. If both fucoidan and EtOH were to be administered at the same time, there was a more than 30-minute dosing interval. To reduce the daily variation of treatment, the same person performed the intragastric delivery of EtOH or fucoidan. After the designed treatment, animal weight was determined. Mice were euthanized by cervical dislocation. Blood samples were collected by penetrating the retro-orbital sinus with a glass capillary tube (0.5 mm in diameter), and serum was obtained after centrifugation (2500× *g*, 30 min).

Serum ALT and AST activities were measured with ALT Enzymatic Assay Kits and AST Enzymatic Assay Kits (Asan Pharm. Co., Korea) following the manufacture’s procedures. Liver, spleen and thymus were carefully removed, and each tissue was weighed after removing as much blood as possible with paper towel.

### 3.5. HepG2 Cell Culture and Treatment with Alcohol and Fucoidan

HepG2 cells (human hepatoma cell line, ATCC HB-8065) were cultured in Dulbecco’s Modified Eagle’s Medium (DMEM) with high glucose supplemented with 10% fetal bovine serum (FBS) and 1% penicillin/streptomycin (100 units/mL penicillin and 100 μg/mL streptomycin; Gibco, Carlsbad, CA, USA) until confluence. Cells were seeded in a 100-mm cell culture dish and pretreated with fucoidan (30 and 60 μg/mL) for 2 h and then stimulated with EtOH (100 mM) for 24 h.

### 3.6. Measurement of Nitric Oxide Production

After treatment with 100 mM EtOH and fucoidan, nitric oxide was measured as nitrite released from HepG2 cells, as previously described [43]. Briefly, 100 µL of supernatant was combined with an equal volume of Griess reagent (1% sulfanilamide, 0.1% naphthalenediamine dihydrochloride, 2.5% phosphoric acid) and incubated at room temperature for 10 min. The absorbance at 540 nm was determined with an E MAX precise microplate reader (Molecular Devices, Eugene, OR, USA), and nitrite concentrations were calculated from a nitrite standard curve.

### 3.7. Protein Extraction and Immunoblot

Protein extraction and immunoblot were performed as previously described [45]. Briefly, the cells or isolated liver tissue were washed twice with cold Dulbecco’s Phosphate-Buffered Saline (D-PBS) and then homogenized in the presence of radio immunoprecipitation assay (RIPA) buffer (25 mM Tris-HCl pH 7.6, 150 mM NaCl, 1% NP-40, 1% sodium deoxycholate, 0.1% sodium dodecyl sulfate (SDS), including protease/phosphatase inhibitor cocktails; Sigma). Equal amounts of protein (50 μg) were electrophoresed on 10% or 12% SDS-polyacrylamide gels and transferred to an Immobilon^®^-P polyvinylidene difluoride membrane, and binding of each specific antibody was visualized using the enhanced chemiluminescence method (Amersham Biosciences, Pittsburgh, PA, USA). Equal loading of samples was confirmed by re-probing the membranes with anti-β-actin antibody. The band density from immunoblots was analyzed using Multi Gauge Ver. 3.0 software (Fujifilm, Tokyo, Japan).

### 3.8. Statistics

All values are expressed as the mean ± SD of at least three independent experiments. Significance was determined using one-way ANOVA followed by Dunnett’s method (Systat Software Inc., San Jose, CA, USA), and *p* < 0.05 was considered significant.

## 4. Conclusions

In this study, the beneficial effects of fucoidan on alcohol-induced liver damage in mice were evaluated. Following alcohol feeding to mice, there were significant increases in liver enzyme aspartate (AST) and alanine (ALT) transaminases. However, fucoidan administration prevented the alcohol-induced increase of these liver enzymes. The critical fibrogenic mediator, TGF-β1, was highly expressed in both the liver of alcohol-fed mice and *in vitro* in HepG2 cells. In both cases, this upregulation of TGF-β1 was strongly suppressed by fucoidan supplementation. The augmented expression of pro-inflammatory COX-2 and NO production by alcohol exposure was also markedly suppressed by fucoidan treatment in mice and HepG2 cells. In addition to reducing proinflammatory mediators, fucoidan enhanced the expression of hepatoprotective HO-1 in both mice and HepG2 cells. HO-1 acts as an antioxidant and contributes to inhibiting liver inflammatory mediators, including iNOS and COX-2. TGF-β1 and COX-2, which have been widely considered to be the most important inflammatory and fibrogenic cytokines in alcohol-induced liver injury. Our results showed that fucoidan supplementation protects against ethanol-induced liver injury, possibly through suppressing hepatic production of the inflammatory cytokines, such as TGF-β1, COX-2 and NO, and enhancing the oxidant defense systems via the activation of the HO-1 pathway. It is unclear whether these determined effects of fucoidan are working separately or together to protect liver cells. Therefore, the protective role of fucoidan on alcohol-induced liver damage and its underlying mechanisms should be further investigated.

Collectively, our findings suggest that fucoidan from *Fucus vesiculosus* is a potential candidate for attenuating alcohol-induced liver damage.
